# Blockley Hospital—Service of W. W. Gerhard, M. D.

**Published:** 1842-06-11

**Authors:** M. W. Wilson

**Affiliations:** Resident Physician


### MEDICAL EXAMINER.

NEW SERIES.

No. 24.] PHILADELPHIA, JUNE 11, 1842. [Vol. I.

### CLINICAL REPORTS.

### *Blockley Hospital—Service of W. W. Gerhard, M.D.*

Reported by M. W. Wilson, M. D., Resident Physician.

#### *Case of Typhoid Erysipelas.*

Joshua Beard, æt. 77, has been an assistant in the ward for fifteen months; for a fortnight has been indisposed; no pain, but felt weak; no appetite; no diarrhœa. Had two chills, one on the 20th, and one on the 24th. Took to his bed on the 20th, and dates his attack from it. Has had no pain except cephalalgia, on the 24th, 25th and 26th, which was very severe. No pain in any of his limbs. There is an old ulcer of many years standing on his leg, which has become red since his attack. Took a dose of oil on the 25th, and on the 26th Mass. Hydrarg. gr. xij., Rhei. Pulv. gr. x., which purged him freely; stools thin and yellow after the oil. Remained in the same prostrate condition since taken; complexion dusky red; pulse frequent and full; appetite lost. Face naturally flushed.

27th. *State.*—Flush of face deeper than natural; tongue white and thick; no appetite; no tenderness; hearing as in health; strength very feeble, (most prominent symptom;) sleep, at first intercepted, is now wanting; no alteration, except feebleness. Pulse 108, feeble, easily compressed; skin warm; no abdominal tenderness. From above the bellies of the muscles of the left leg erysipelatous redness of skin, of a purplish tint, extending downwards to the instep; circulation in toes feeble, cool; skin flaccid. Ordered flaxseed mucilage to leg.

℞. Vini ℥iv. per diem.

℞. Infus. Serpentar.

℞. Acet. Ammon. ℥ss. q. h. s.

℞. Hydrarg. cum creta. gr. xij.

P. Ipecac. et Opii. gr. v.

Camphoræ, gr. ij.

M. At night.

28th. Redness extending slowly both up and down; no change in other respects; bowels not moved for two days. Ordered a common injection. Continue treatment, except mercurial and anodyne at night.

29th. Tongue moist and clean; pulse soft and feeble, 104; appetite good; countenance improved; redness diminishing; more of a rose colour over the foot. Continue treatment.

30th. Redness extending; is now above the knee; more painful; had rigors this morning; tongue dry, and coated in centre; expression more anxious; appetite failing. Pulse 100, weak; bowels open once last night. Continue treatment.

May 1st. Redness extending very slowly; pulse 108, feeble; appetite somewhat better; bowels opened several times since. Continue treatment.

2d. Skin cool; pulse regular, 60; redness as yesterday.

℞. Emplas. Epispas. around the thigh above the redness. Continue other treatment.

4th. Swelling and redness better; colour brighter; capillaries more active; redness extends to the blister, but not above it; skin cool; pulse regular 85; tongue cleaning. Continue treatment.

5th. Much improved; feels better.

6th. Redness diminishing; skin cool; pulse 80, soft and full; appetite good. An abscess formed on the foot, and discharged about three-fourths of an ounce of pus. Continue treatment.

8th. Redness disappearing, now of a pale red colour; tongue nearly clean; appetite good. In all respects better. Continue treatment.

11th. Continues improving; appetite good; tongue natural; abscess on foot discharging a little yet; redness fading.

14th. Continues improving; redness almost gone; foot still a little swelled; feels well; appetite good.

18th. Quite well, except somewhat weak. Strength is improving very fast.

[Erysipelas has prevailed to an unusual extent during the past spring in the Philadelphia hospital, and with it the usual attendant, puerperal fever. The present case is more interesting than usual, because the humoral character of the disease was very apparent from the beginning. The extreme prostration, the dusky tint of the skin, and the stupid condition of the patient, were the first symptoms, and were noticed before the attention was called to the erysipelas. It was afterwards ascertained that the patient had been dull and feverish for a week or fortnight previously to his attack. At the time there were many erysipelatous patients in the wards, upon whom he was compelled to be in constant daily attendance, sleeping in the same apartment with them.

The typhoid state of this case of erysipelas was very similar to that of the typhus fever patients who were in the wards about the same time. The same cause evidently existed in both cases; that is, an altered state of the blood. The distinctive characters of typhus depend, therefore, not upon what are termed the typhoid symptoms, but upon the eruption, and the succession of phenomena.

The blister was successful in arresting the erysipelas; where it can be used it will be found to be a much more certain application than the nitrate of silver; but local treatment would have been useless without the constitutional remedies which were required.—W. W. G.]

